# Morphological changes in the cerebellum during aging: evidence from convolutional neural networks and shape analysis

**DOI:** 10.3389/fnagi.2024.1359320

**Published:** 2024-04-17

**Authors:** Yu Wang, Ye Teng, Tianci Liu, Yuchun Tang, Wenjia Liang, Wenjun Wang, Zhuoran Li, Qing Xia, Feifei Xu, Shuwei Liu

**Affiliations:** ^1^Department of Anatomy and Neurobiology, Research Center for Sectional and Imaging Anatomy, Shandong Provincial Key Laboratory of Mental Disorder, Shandong Key Laboratory of Digital Human and Clinical Anatomy, School of Basic Medical Sciences, Cheeloo College of Medicine, Shandong University, Jinan, China; ^2^Institute of Brain and Brain-Inspired Science, Shandong University, Jinan, China; ^3^Department of Ultrasound, Shandong Provincial Hospital Affiliated to Shandong First Medical University, Jinan, China

**Keywords:** aging, cerebellar atrophy, shape analysis, convolutional neural networks, sex difference

## Abstract

The morphology and function of the cerebellum are associated with various developmental disorders and healthy aging. Changes in cerebellar morphology during the aging process have been extensively investigated, with most studies focusing on changes in cerebellar regional volume. The volumetric method has been used to quantitatively demonstrate the decrease in the cerebellar volume with age, but it has certain limitations in visually presenting the morphological changes of cerebellar atrophy from a three-dimensional perspective. Thus, we comprehensively described cerebellar morphological changes during aging through volume measurements of subregions and shape analysis. This study included 553 healthy participants aged 20–80 years. A novel cerebellar localized segmentation algorithm based on convolutional neural networks was utilized to analyze the volume of subregions, followed by shape analysis for localized atrophy assessment based on the cerebellar thickness. The results indicated that out of the 28 subregions in the absolute volume of the cerebellum, 15 exhibited significant aging trends, and 16 exhibited significant sex differences. Regarding the analysis of relative volume, only 11 out of the 28 subregions of the cerebellum exhibited significant aging trends, and 4 exhibited significant sex differences. The results of the shape analysis revealed region-specific atrophy of the cerebellum with increasing age. Regions displaying more significant atrophy were predominantly located in the vermis, the lateral portions of bilateral cerebellar hemispheres, lobules I-III, and the medial portions of the posterior lobe. This atrophy differed between sexes. Men exhibited slightly more severe atrophy than women in most of the cerebellar regions. Our study provides a comprehensive perspective for observing cerebellar atrophy during the aging process.

## 1 Introduction

The human cerebellum lies in the posterior cranial fossa and is composed of a core of white matter and a superficial cortex. The cerebellar cortex is characterized by a high degree of convolution, resulting in the formation of lobes and lobules. These lobes and lobules are then subdivided into smaller leaf-like structures called folia (Goldwyn, 1985). Accumulating evidence indicates progressive cerebellar atrophy with age (Luft et al., 1999; Hulst et al., 2015; Steele and Chakravarty, 2018; Han et al., 2020). Nevertheless, abnormal atrophy of the cerebellum can result in various neurological impairments, including movement coordination issues (Abdelgabar et al., 2019; Cortese et al., 2020), memory deterioration (Klockgether et al., 2019), and speech dysfunctions (Rusz et al., 2019).

The significance of the cerebellum has led to extensive investigations into its aging trajectory. Studies have confirmed a reduction in the volume of the vermis with age (Luft et al., 1999; Koppelmans et al., 2017). Similarly, the volume of bilateral Crus I decreases gradually during normal aging (Bernard and Seidler, 2013; Koppelmans et al., 2017). Longitudinal research by Raz et al. (2013) and Han et al. (2020) further revealed shrinkage in certain cerebellar subregional volumes with age. While the majority of these studies focused on aging trends in cerebellar subregional volumes, providing valuable insights into volumetric changes throughout the aging process, they offered limited information on morphological alterations within different cerebellar regions. Shape analysis, however, can be used to identify local alterations occurring through development or the aging process in brain regions (Shi et al., 2014; Ge et al., 2021). Consequently, this study aimed to evaluate the morphological changes in the cerebellum from different perspectives by integrating results from local volume measurements and shape analysis.

Previous research has shown that distinct regions within the cerebellum are involved in motor (Mottolese et al., 2013), cognitive (Guell et al., 2018), and emotional processes (Stoodley and Schmahmann, 2018; Schmahmann, 2019). Furthermore, neuroimaging studies have elucidated that particular sensorimotor tasks and advanced cognitive functions are associated with specific lobules within the cerebellum (Diamond, 2000). For instance, a functional neuroimaging study revealed activation related to motor tasks in lobules IV, V, VI, and VIII, whereas cognitive tasks elicited activation in lobules VI, VIIB, IX, and X, Crus I, and Crus II (Guell et al., 2018). The functional specificity of cerebellar regions was further corroborated by observations in patients with cerebellar lesions. Lesions in the anterior lobe of the cerebellum can result in characteristic cerebellar motor syndromes, such as gait ataxia, limb dyskinesia, and dysarthria (Schmahmann et al., 2012). Lesions in the posterior lobe of the cerebellum have been associated with the onset of cerebellar cognitive affective syndrome (CCAS), yet they do not significantly impact motor functions (van Harskamp et al., 2005; Stoodley et al., 2016). In neurodegenerative diseases such as Alzheimer’s disease and Parkinson’s disease, the cerebellum is a critically impacted region, with morphological alterations over time due to sustained damage (Iskusnykh et al., 2024). Consequently, examination of cerebellar regional changes in individuals who are cognitively normal during the aging process can provide valuable insights into the pathology of neurodegenerative disorders, such as Alzheimer’s disease and Parkinson’s diseases. Furthermore, a detailed exploration of cerebellar atrophy patterns inherent to normal aging processes is instrumental for guiding the development of precise therapeutic interventions.

To comprehensively understand cerebellar atrophy associated with aging, we utilized convolutional neural networks and shape analysis to investigate the morphological changes in the cerebellum in a cohort of 553 healthy individuals aged 20 to 80 years. These methodologies were complementary and collectively supported a detailed assessment of the structural changes in the cerebellum attributable to aging. By investigating cerebellar morphology from both global and regional perspectives, our study aimed to explore the morphological changes in the cerebellum during the aging process.

## 2 Materials and methods

### 2.1 Dataset and participants

Structural T1-weighted MR images from the Information eXtraction from Images (IXI) database (https://brain-development.org/ixi-dataset/) were used in this study. The dataset is publicly available and includes 588 healthy subjects (after quality control, *N* = 553). The images were collected with 1.5 T and 3 T scanners (FOV = 256 mm × 256 mm, matrix size = 0.9375 × 0.9375 × 1.2 mm^3^) at 3 sites. Demographic information is provided in Table 1.

**Table 1:** Demographic information.

Group (y)	Sex (male/female) (after QC)	Age (mean ± SD)	Total
20–29	45/56	(25.15 ± 2.75)/(25.25 ± 2.70)	101
30–39	60/39	(34.08 ± 2.90)/(34.38 ± 2.54)	99
40–49	41/48	(44.29 ± 2.77)/(43.98 ± 2.80)	89
50–59	38/60	(55.03 ± 2.82)/(55.37 ± 2.80)	98
60–69	47/70	(64.72 ± 3.02)/(63.69 ± 2.88)	117
70–79	15/34	(73.07 ± 2.59)/(72.73 ± 2.31)	49

### 2.2 Image processing

**Figure 1:**
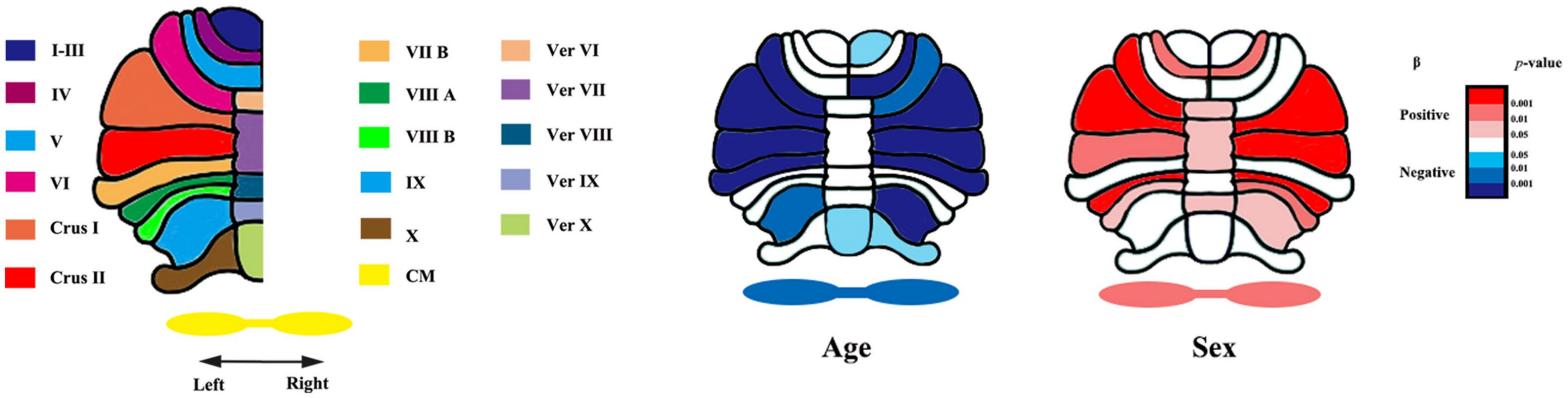
Segmentation of cerebellar subregions and raw *p*-values of age and sex for each region. Ver, vermis. CM, corpus medullare. β, fixed coefficients. β <0 indicates that absolute volume decreases with increasing age. β >0 indicates males having larger cerebellar subregion’s absolute volumes than females.

We applied convolutional neural networks algorithm (ACAPULCO) to segment cerebellar subregions. Compared to other methods (Diedrichsen, 2006; Chakravarty et al., 2013; Weier et al., 2014; Romero et al., 2017), this approach can significantly improve the speed of segmentation while ensuring high accuracy. The algorithm consists of the following steps. The original images were inhomogeneity corrected in native space using N4 bias field correction. Then, the images were rigidly registered to the 1 mm isotropic ICBM 2009c template in Montreal Neurological Institute (MNI) space with Advanced Normalization Tools (ANTs) registration suite. Voxel-by-voxel labeling of the preprocessed images was performed using parcellation classifiers trained on manual delineations from 15 experts (Carass et al., 2018). Then, small, isolated fragments were eliminated during postprocessing. Finally, the algorithm identified 28 cerebellar regions, namely, bilateral lobules I–III, IV, V, and VI; Crus I and II; lobules VIIB, VIIIA, VIIIB, IX, and X; vermis VI, VII, VIII, IX, and X; and the corpus medullare (CM) (Figure 1).

Segmentation of the whole cerebellum and estimation of the total intracranial volume (ICV) were performed with FreeSurfer (http://surfer.nmr.mgh.harvard.edu). Then, all cerebellar voxels were extracted for shape analysis. Previous studies confirmed that shape analysis is an excellent method for assessing regional alterations (Xu et al., 2020) and tracking surface aging trajectories in the cerebellar cortex. Shape analysis was performed with the fully automated Metric Optimization for Computational Anatomy (MOCA) software (https://www.nitrc.org/projects/moca_2015/). First, the cerebellum template was constructed using ANTs to improve the accuracy of the shape analysis registration step. Then, the binary mask of each cerebellum and that of each template were transformed into triangular meshes. Iterated Laplace–Beltrami (LB) eigenprojection and boundary deformation (Shi et al., 2014; Ge et al., 2021) were applied to detect and remove the spurious features associated with segmentation artifacts. The resulting surface meshes accurately representing the topology of the cerebellum were remeshed to obtain 6,000 evenly distributed vertices. The number of vertices (6000) was determined according to the size of the cerebellum and the computational cost. Each individual triangulated mesh was registered to the constructed cerebellar template mesh by intrinsic surface mapping in the high-dimensional LB embedding space (Shi et al., 2014).

Ultimately, all surfaces were represented by the same triangulation scheme and vertex-to-vertex correspondence pattern. The quantification of the local rate of aging in the cerebellum was similar to that used for hippocampal shape analysis (Shi et al., 2009), and the thickness measured at each vertex of the mapped surfaces was defined as the distance from the vertex to the medial core of the cerebellum.

### 2.3 Statistical analysis

Using low-order polynomial functions to model the trajectory of structural volume is an approach commonly used in research studies (Tiemeier et al., 2010; Walhovd et al., 2011; Romero et al., 2017). Based on the previous research (Romero et al., 2021), we selected several functions to analyze the volume of each cerebellar subregion during normal aging. The trajectory models are expressed as follows:

Linear model


$$Vol=\beta_{0}+\beta_{1}\text{Age}+\varepsilon$$

Quadratic model


$$Vol=\beta_{0}+\beta_{1}\text{Age}+\beta_{2}Age^{2}+\varepsilon$$

Cubic model


$$Vol=\beta_{0}+\beta_{1}\text{Age}+\beta_{2}Age^{2}+\beta_{3}Age^{3}+\varepsilon$$

Considering the model selection methodology and the insights gleaned from prior research (Han et al., 2020; Romero et al., 2021), linear regression was employed for the analysis of absolute volume, and the relative volume was analyzed based on the results of fitting with a low-order polynomial model. We accounted for sex and the interaction between sex and age as covariates in the analyses.

To assess the local atrophy of the cerebellum, linear regression analysis was applied to detect changes in thickness with age. Sex and the interaction between sex and age were accounted for as covariates. Regarding the sex differences in the shape analysis, we conducted a t test and used the ICV as a covariate. The *p* value and regression coefficient were measured. The false discovery rate (FDR) was used to correct for multiple comparisons. All the statistical analyses were conducted in MATLAB 2018b.

### 2.4 Quality control

First, we included individuals aged between 20 and 80 years. Then, we removed 3 images based on the original image quality. Five images were excluded based on the loss of cerebellar segmentation results in FreeSurfer. Finally, a total of 553 individuals were included in this study after full data control. The specific population information is shown in Table 1.

## 3 Results

A detailed description of the morphological changes in each structure with age is given below. Given that the dataset did not include information on handedness, this factor was not accounted for in our study.

### 3.1 Total cerebellum and corpus medullare

**Figure 2:**
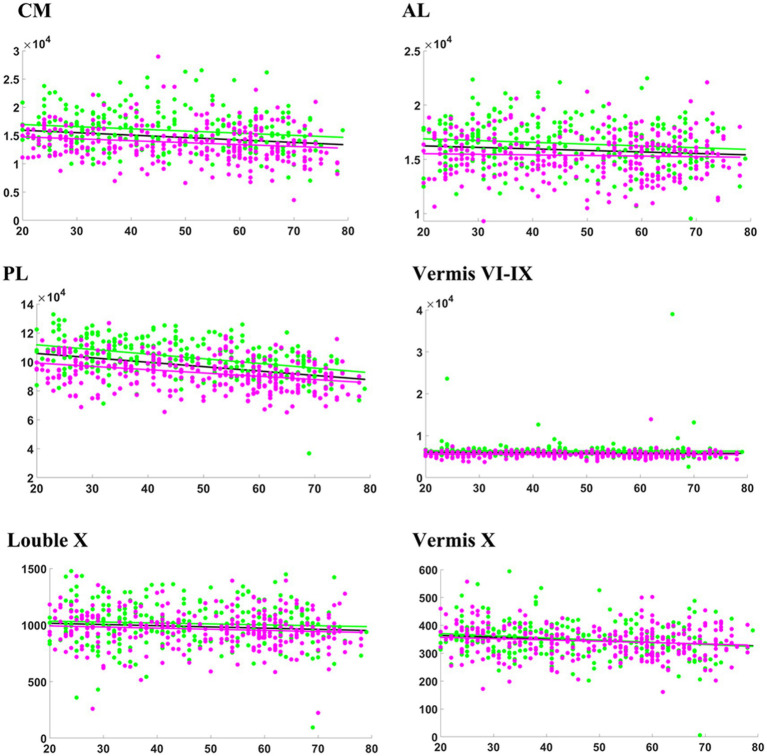
Relationship between absolute volumes (mm^3^) of cerebellar subregions and age. CM, corpus medullare. Anterior lobe (AL) consists of Lobules I-III, IV, and V. Posterior Lobe (PL) comprises of Lobule VI, Crus I, Crus II, and Lobules VIIB, VIIIA, VIIIB, and IX. The general model is depicted in black, while the female model is illustrated in magenta, and the male model in green.

Table 2 presents the results of a linear analysis of the absolute volume of the cerebellar subregions, and Figure 1 provides a visual representation of the original *p* values obtained from Table 2. Neither sex nor the interaction between sex and age significantly affected the absolute volume of the total cerebellum or CM (*p* > 0.05) (Table 2). Figure 2 displays the fitted overall mean trajectories of the cerebellar subregions, including CM, vermis VI-IX, vermis X, as well as anterior lobe, posterior lobe, and flocculonodular lobe (comprised solely of Lobule X) of the cerebellum. The scatter plot results for the absolute volumes of 28 cerebellar subregions are presented in Supplementary Figure S1. An analysis of the relative volumes of the cerebellar subregions is provided in Supplementary Table 1. In terms of absolute volume, the total cerebellum (Supplementary Figure S1) and CM (Figure 2) demonstrated gradual declines (*p* < 0.05). The relative volume of the total cerebellum significantly decreased with age (*p* < 0.05) (Supplementary Table S1). The absolute volumes of the cerebellum and CM were significantly greater in males than in females (*p* < 0.05) (Table 2).

**Table 2:** Linear regression results of the absolute volume of the cerebellar subregions.

Absolute volume	*R* ^2^	Age		Sex		Age × Sex	
		*β*	*p*-value	*β*	*p*-value	*β*	*p*-value
Total	0.554	−269.449	0.000**	19289.501	0.000**	−110.087	0.139
CM	0.125	−33.815	0.004*	2283.891	0.009*	−4.627	0.793
AL	0.074	−5.479	0.453	1590.907	0.004*	−10.928	0.326
L I-III	0.022	−1.133	0.069	38.107	0.420	−0.107	0.910
R I-III	0.028	−1.491	0.026*	51.684	0.307	−0.628	0.536
L IV	0.036	4.494	0.053	472.009	0.008*	−4.937	0.163
R IV	0.064	2.170	0.401	656.604	0.001*	−6.052	0.125
L V	0.041	−2.206	0.224	244.732	0.076	−1.302	0.638
R V	0.094	−7.313	0.000**	127.731	0.345	2.099	0.439
PL	0.294	−228.169	0.000**	14475.056	0.000**	−93.222	0.113
L VI	0.215	−25.886	0.000**	1522.130	0.000**	−11.692	0.124
R VI	0.083	−20.767	0.002*	792.653	0.127	2.320	0.824
L Crus I	0.208	−36.715	0.000**	2010.931	0.000**	−16.801	0.127
L Crus II	0.136	−21.907	0.000**	1003.691	0.008*	−6.452	0.397
L VIIB	0.086	−21.792	0.000**	305.152	0.428	4.866	0.529
R Crus I	0.192	−30.860	0.000**	2241.760	0.000**	−20.338	0.057
R Crus II	0.225	−23.357	0.000**	1983.589	0.000**	−18.898	0.018*
R VIIB	0.090	−20.620	0.000**	−42.201	0.882	6.605	0.248
L VIIIA	0.233	−4.342	0.369	1869.123	0.000**	−8.782	0.234
LVIIIB	0.046	2.501	0.257	409.507	0.015*	−2.886	0.390
R VIIIA	0.109	−6.385	0.133	1219.265	0.000**	−10.934	0.092
R VIIIB	0.051	−3.500	0.208	550.128	0.009*	−5.857	0.167
L IX	0.051	−6.595	0.003*	225.065	0.186	−1.530	0.654
R IX	0.094	−7.910	0.000**	391.679	0.019*	−3.698	0.267
FL	0.026	−0.992	0.114	37.945	0.425	0.180	0.850
L X	0.017	−0.153	0.670	20.150	0.460	0.130	0.812
R X	0.032	−0.837	0.014*	17.849	0.487	0.049	0.924
Vermis VI-IX	0.050	−0.655	0.918	874.592	0.072	−1.045	0.915
Vermis VI	0.042	0.173	0.842	159.304	0.016*	−1.267	0.339
Vermis VII	0.057	0.525	0.448	123.946	0.019*	−0.586	0.100
Vermis VIII	0.021	−0.369	0.940	162.381	0.663	5.092	0.495
Vermis IX	0.027	−0.979	0.726	429.236	0.044*	−4.290	0.315
Vermis X	0.032	−0.480	0.021*	19.403	0.220	−0.304	0.338

Total, cerebellar volume; CM, corpus medullare; AL, anterior lobe; PL, posterior lobe, L, left; R, right. Age × Sex represents the interaction between age and sex. **p < 0.001; *p < 0.05.

### 3.2 Anterior lobe and lobules

Lobules I-III, IV, and V constitute the anterior lobe (AL). The AL gradually decreased in absolute volume with increasing age (Figure 2). Regression analysis indicated that the absolute volume of right lobules I-III and V significantly decreased with age (Table 2). The relative volumes of bilateral lobule IV displayed a significant aging trend (Supplementary Table 1). In Table 2, *β* < 0 indicates a decrease in volume with increasing age and that males exhibit larger volumes in the subregions of the cerebellum than females with increasing age. Specifically, compared with females, males exhibited a greater absolute volume of AL (*β* = 1590.907, *p* = 0.004). Significant sex differences were found for bilateral lobule IV (*β*_l_ = 472.009, *p*_l_ = 0.008; *β*_r_ = 656.604, *p*_r_ = 0.001). According to the analysis of relative volume, only right lobule V exhibited a significant sex-related difference (*p* = 0.033) (Supplementary Table S1). Specifically, males exhibited a larger absolute volume for bilateral lobule IV and a greater relative volume for right lobule V than females.

### 3.3 Posterior lobe and lobules

The posterior lobe (PL) comprises lobule VI, Crus I, Crus II, and lobules VIIB, VIIIA, VIIIB, and IX. The absolute volume of the PL significantly and gradually decreased with increasing age (Figure 2; Table 2). The regression analysis results indicated a significant decrease in the absolute volumes of the bilateral lobule VI, Crus I, Crus II, and lobules VIIB and IX with age (Table 2). The absolute volumes of the bilateral Crus I, Crus II, and lobules VIIIA and VIIIB displayed significant sex differences. Furthermore, the absolute volumes of left lobule VI and right lobule IX also exhibited significant sex differences (Table 2). The absolute volumes of these regions (bilateral Crus I, Crus II, and lobules VIIIA and VIIIB; left lobule VI; and right lobule IX) were greater for males than females. For right Crus II, the relation between age and sex was significant, with a negative coefficient indicating a faster decline in men than in women (*p* = 0.018). As shown in Supplementary Table S1, there was a significant sex-related difference in the relative volumes of the right Crus I, Crus II, and lobule VIIB. The relative volume of the PL also exhibited a significant aging trend. Furthermore, the relative volumes of the left lobules VI and VIIIA, bilateral Crus I and Crus II, and right lobules VIIB and VIIIB were significantly correlated with age. The relative volumes of the right Crus II, lobule VIIB and left lobule VIIIA exhibited significant differences based on sex. Males showed significantly lower relative volume in the right Crus II and lobule VIIB compared to women. Additionally, males demonstrated a significantly greater relative volume in the left Lobule VIIIA.

### 3.4 Flocculonodular lobes

From the scatter plot, we observed a gradual decreasing trend in the absolute volume of lobule X. According to the regression analysis results (Table 2), only right lobule X exhibited a significant age-related declining trend (*p* < 0.05).

### 3.5 Vermis

For the vermis, volume changes associated with sex and the relation between sex and age were not statistically significant (*p* > 0.05) (Table 1). Vermis X showed a significant decreasing trend in absolute volume (*p* < 0.05). There were significant sex differences in the absolute volumes of vermis VI, VII and IX (*p* < 0.05) (Figure 2). Compared to females, males exhibited greater absolute volumes of vermis VI, VII, and IX. In addition, vermis VI displayed a significant aging trend in terms of relative volume (Supplementary Table S1).

### 3.6 Shape analysis

**Figure 3:**
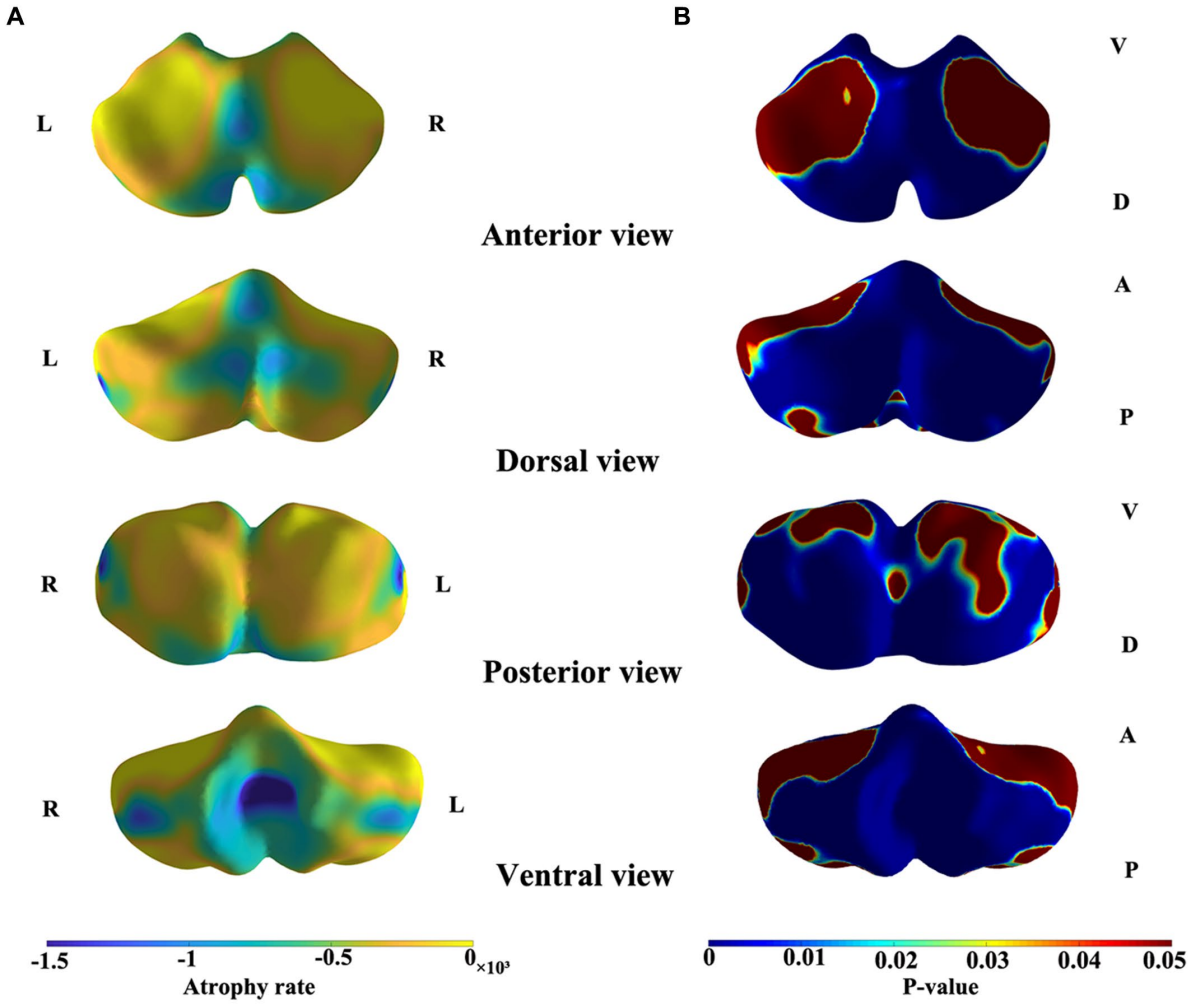
Shape atrophy of the cerebelum with increasing age. Color gradients in panel A represent different degrees of atrophy. Color gradients in panel B represents the *p*-values after FDR correction. A, anterior, P, posterior, D, dorsal, V, ventral. **(A)** Blue indicates higher atrophy rates and yellow indicates lower atrophy rates. **(B)** Blue indicates lower *p*-values and red indicates higher *p*-values.

**Figure 4:**
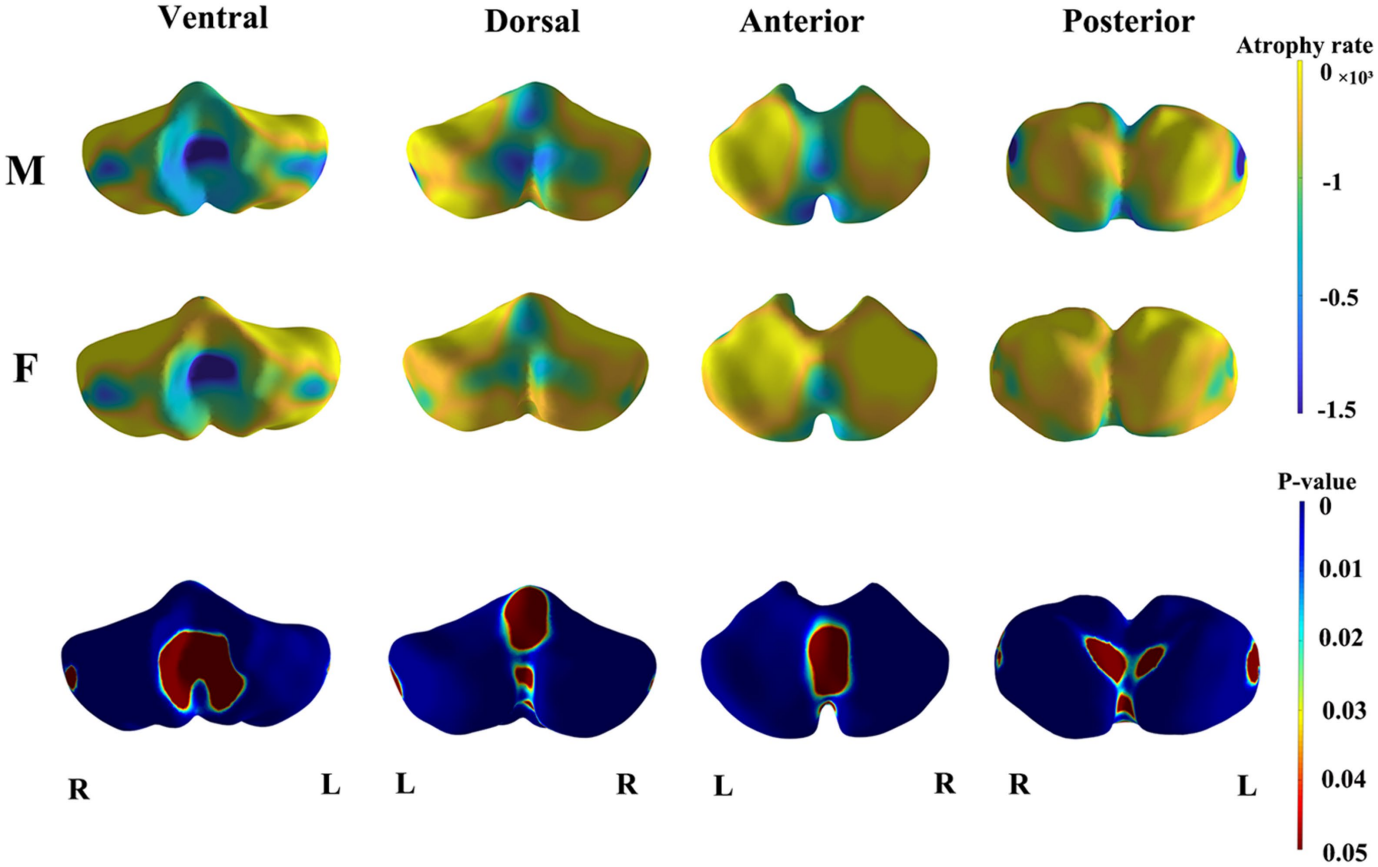
The results of sex differences in cerebellar morphology. The first two rows demonstrate the age-related trends of cerebellar atrophy in different genders (M, Male; F, Female). Blue indicates higher atrophy rates and yellow indicates lower atrophy rates. The third row displays the *p*-values after FDR correction for the gender differences in cerebellar thickness. Blue indicates lower *p*-values and red indicates higher *p*-values.

The results of the shape analysis, which considered sex and the interaction between sex and age, indicated that the interaction between sex and age was not statistically significant. Figures 3 and 4 illustrate the mapping of the shape analysis results onto the template surface of the cerebellum. The local cerebellar atrophy rate was represented by the regression coefficients (Figure 3A), and the corresponding thickness of the 6,000 vertices was explored with linear regression analysis to calculate *p*-values (Figure 3B). The different colors overlaying the template in Figure 3A represent different atrophy rates, with blue indicating higher atrophy rates and yellow indicating lower atrophy rates. In Figure 3B, the different colors represent the magnitudes of the *p* values, with blue indicating lower *p*-values and red indicating higher *p* values. Figure 3 illustrates the trend of overall cerebellar atrophy with increasing age, characterized by varying degrees of atrophy in different regions. Specifically, atrophy is markedly more pronounced in the vermis, the lateral sections of the bilateral cerebellar hemispheres, lobules I-III, and the medial sections of the posterior lobe than in other regions. Figure 4 illustrates the differences in cerebellar atrophy between different sexes. From the first two rows of Figure 4, it can be observed that there are differences in cerebellar atrophy between males and females in most regions. The third row shows the *p* values obtained from *t* tests, which have been corrected for FDR. From the four views, it can be observed that there are significant sex differences in the thickness of most regions of the cerebellum, except for most parts of the vermis and some parts of the cerebellar hemispheres.

## 4 Discussion

In our research, we utilized volumetric and morphological analyses to assess the progression of cerebellar atrophy with advancing age. We observed a marked reduction in the total absolute volume of the cerebellum, consistent with findings from previous studies (Luft et al., 1999; Sullivan et al., 2000; Raz et al., 2005; Romero et al., 2021). Notably, the effects of aging on various cerebellar subregions are heterogeneous (Han et al., 2020). Specifically, the results demonstrated a significant age-related decline in the absolute volumes of CM, AL, PL, lobule X, and vermis X (Figure 2). The results of shape analysis further demonstrated that atrophy in different regions of the cerebellum varies with age (Figure 3).

Our investigation revealed significant differences in the subregional absolute volume of the cerebellum. The cerebellar parcellation algorithm based on convolutional neural networks was effective in the segmentation of 28 cerebellar subregions. The analysis indicated a statistically significant reduction in the absolute volumes of 15 subregions with advancing age. The results of our investigation slightly diverge from those reported by Han et al. (2020), despite using the same segmentation method. We speculate that this discrepancy may be due to the wider age range of our sample, variations in sample characteristics and the use of different statistical methods. Our study revealed a significant age-related trend, specifically in the right lobules I-IV and V. A comparative study of voxel-based morphometry (VBM) in cerebellar patients and healthy individuals revealed that the pattern of cerebellar degeneration with age was similar, but not identical, to that of patients with cerebellar degenerative disease. The VBM results of the study indicated that the anterior cerebellar lobe (lobules I-V) exhibited the most pronounced cerebellar volume reduction in both patient datasets (Hulst et al., 2015). This study provides strong support for our results. The absolute volumes of bilateral Crus I and lobule VI showed a significant age-related declining trend, which was supported by the results of Ramanoel et al., who found substantial atrophy in Crus I among older adults using the SUIT toolbox (Ramanoel et al., 2018). In contrast to lobules I-VI, which primarily facilitate the coordination and fine regulation of bodily movements, lobule VI/Crus I predominantly participates in executing complex cognitive tasks, such as those associated with language processing and working memory (Guell et al., 2018). We hypothesize that morphological alterations in bilateral Crus I and lobule VI associated with aging will impact working memory functions, among others. The precise nature of the relationship between structural changes and functional outcomes necessitates further experimental exploration. In this study, bilateral Lobule VIIB demonstrate age-related decline and this decline is statistically significant. Lobule VII is a secondary sensorimotor structure (Debas et al., 2010). The left cerebellar lobule VIIB is involved in executive functioning, with impairments in this domain correlating with an elevated risk of pervasive psychopathology among children and adolescents (Buckner et al., 2011). Our study also revealed a significant reduction in the absolute volume of bilateral lobule IX with aging. A study of the cerebellum and cognition in Friedrich’s ataxia patients showed a direct correlation between the volume of lobule IX and impaired visual spatial function (Cocozza et al., 2020). Such findings may elucidate the reduction in visual spatial abilities observed in the elderly population. Lobule X mainly participates in maintaining balance, posture, and eye movements (Schniepp et al., 2017). The absolute volume of the right lobule X showed a significant age-related decrease. The impact of aging on the function of the right lobule X should be further investigated.

Several investigations have reported a decrease in the volume of the vermis with advancing age (Luft et al., 1999; Koppelmans et al., 2017). However, our study suggested that only the absolute volume reduction in vermis X exhibited a significant age-related decreasing trend. With respect to changes in the absolute volume of the vermis during the aging process, existing research does not provide a uniform conclusion (Bernard and Seidler, 2013; Bernard et al., 2015; Han et al., 2020). Variations in datasets, statistical methodologies, segmentation protocols, and analytical techniques may account for the discrepancies observed among the findings.

Our study revealed statistically significant sex-related variations in the absolute volumes of 16 out of the 28 segmented subregions in the cerebellum. This finding aligns with previous studies confirming cerebellar structural sexual dimorphism (Fan et al., 2010; Bernard et al., 2015). It is widely accepted that the brains of males tend to be larger than those of females within the same age group (Good et al., 2001; Luders and Kurth, 2020; Joel, 2021). Consequently, we analyzed an analysis of the normalized cerebellar volumes, as detailed in our Supplementary Materials. This analysis revealed significant sex-based differences in the relative volumes of only 4 out of the 28 cerebellar subregions examined. Contrary to our findings, Romero et al. did not detect statistically significant sex-based differences in the relative volumes of cerebellar subregions, despite noting significant differences in their absolute volumes (Romero et al., 2021). We postulate that the disparity between our study and Romero’s study is a result of our use of a more detailed segmentation approach and different sample sizes. Shape analysis results further suggested that while cerebellar atrophy with advancing age exhibits regional specificity in both sexes, the degree of atrophy varies between sexes, with male cerebellums showing marginally more pronounced atrophy than those of female (Figure 4).

Unlike previous studies (Fan et al., 2010; Bernard and Seidler, 2013; Bernard et al., 2015; Han et al., 2020), our research presents corroborative evidence of progressive cerebellar atrophy throughout the aging process obtained with two complementary approaches. While regional volume measurements provide a conventional quantitative description of cerebellar aging, shape analysis further characterizes cerebellar atrophy with age from an intuitive perspective, utilizing the thickness values of cerebellar surface vertices. This dual approach contributes to a closer understanding of the cerebellum ageing trajectories over time, with potential applications in neurodegenerative diseases.

Our study has several limitations. The smoothing steps inherent in shape analysis processing may obscure the visibility of cerebellar sulci on the surface, complicating the precise delineation of cerebellar subregions. Furthermore, neural network algorithms are predominantly used to assess the local volume of the cerebellum, in contrast to shape analysis, in which the cerebellum’s thickness, as defined by its vertices, is evaluated. Consequently, the correlation between these two methodologies is somewhat limited. Nonetheless, the application of shape analysis was intended to validate the progressive cerebellar atrophy associated with aging. Therefore, the integration of these methodologies is valuable. Given its cross-sectional design, this study inherently lacks the capacity to explore temporal changes at the individual level over time. Moreover, the diversity of sequence acquisition in publicly available datasets, coupled with the restricted clinical information on the subjects included and the absence of cerebrovascular quantification for the participants, could potentially influence the study’s findings, a prevalent challenge commonly encountered with the use of such datasets. Addressing such issues remains an essential future task.

In conclusion, in our research, cerebellar regional volume changes and the extent of cerebellar cortical atrophy across a normal population aged between 20 and 80 years were systematically examined. We identified region-specific atrophy within the cerebellum and found that it intensifies with age. Furthermore, our findings revealed that males experienced more pronounced atrophy in certain cerebellar regions than did females. This study provides comprehensive insights into cerebellar atrophy during normal aging and a structural basis for identifying potential therapeutic targets and devising strategies to address abnormal cerebellar atrophy.

## Data availability statement

The datasets presented in this study can be found in online repositories. The names of the repository/repositories and accession number(s) can be found in the article/Supplementary material.

## Ethics statement

The studies involving humans were approved by https://brain-development.org/ixi-dataset/. The studies were conducted in accordance with the local legislation and institutional requirements. The participants provided their written informed consent to participate in this study. Written informed consent was obtained from the individual(s) for the publication of any potentially identifiable images or data included in this article.

## Author contributions

YW: Conceptualization, Data curation, Investigation, Methodology, Software, Writing – original draft, Writing – review & editing. YeT: Writing – review & editing. TL: Data curation, Investigation, Methodology, Software, Supervision, Writing – review & editing. YuT: Writing – review & editing. WL: Software, Writing – review & editing. WW: Investigation, Software, Writing – review & editing. ZL: Writing – review & editing, Supervision. QX: Writing – review & editing, Supervision. FX: Methodology, Software, Writing – original draft, Writing – review & editing. SL: Writing – original draft, Writing – review & editing, Supervision.
