# Supplementary material for: Morphological changes in the cerebellum during aging: evidence from convolutional neural networks and shape analysis
Source: Front Aging Neurosci. 2024 Apr 17;16:1359320. doi: 10.3389/fnagi.2024.1359320 (PMC11061448; doi:10.3389/fnagi.2024.1359320)
Supplement: Supplementary file 1 [file Table_1.docx]

Supplementary table 1 Selection of the model and statistical analysis for relative volume measurements.

| Relative volume | Selected  mode | F-statistic | R^2^ | Age | Sex | Age×Sex |
| --- | --- | --- | --- | --- | --- | --- |
|  |  |  |  | *p* | *p* | *p* |
| Total | Second order | 14.178 | 0.094 | 0.002* | 0.680 | 0.046 |
| CM | Third  order | 3.315 | 0.029 | 0.240 | 0.542 | 0.494 |
| AL | linear | 10.220 | 0.069 | 0.202 | 0.598 | 0.146 |
| L I-III | Third  order | 7.284 | 0.062 | 0.387 | 0.191 | 0.616 |
| R I-III | Third  order | 8.977 | 0.076 | 0.357 | 0.267 | 0.340 |
| L IV | linear | 12.847 | 0.066 | 0.000* | 0.760 | 0.640 |
| R IV | linear | 4.974 | 0.026 | 0.014* | 0.452 | 0.084 |
| L V | Third  order | 7.298 | 0.063 | 0.161 | 0.286 | 0.402 |
| R V | Two order | 8.117 | 0.056 | 0.480 | 0.033* | 0.655 |
| PL | Two order | 16.449 | 0.107 | 0.005* | 0.627 | 0.048 |
| L VI | linear | 14.921 | 0.075 | 0.001* | 0.521 | 0.095 |
| R VI | Two order | 2.269 | 0.016 | 0.114 | 0.469 | 0.888 |
| L Crus I | linear | 16.568 | 0.083 | 0.001* | 0.886 | 0.098 |
| L Crus II | Two order | 9.363 | 0.064 | 0.014* | 0.843 | 0.318 |
| L VIIB | Two order | 4.391 | 0.031 | 0.410 | 0.160 | 0.492 |
| R Crus I | linear | 13.464 | 0.069 | 0.032* | 0.550 | 0.038 |
| R Crus II | Two order | 12.583 | 0.084 | 0.016* | 0.034* | 0.009 |
| R VIIB | linear | 18.039 | 0.090 | 0.000* | 0.003* | 0.360 |
| L VIIIA | Two order | 11.131 | 0.075 | 0.026* | 0.001* | 0.159 |
| LVIIIB | Third  order | 3.666 | 0.032 | 0.213 | 0.631 | 0.196 |
| R VIIIA | Two order | 2.945 | 0.020 | 0.075 | 0.051 | 0.075 |
| R VIIIB | Two order | 3.684 | 0.026 | 0.016* | 0.422 | 0.107 |
| L IX | Third order | 7.148 | 0.061 | 0.641 | 0.290 | 0.487 |
| R IX | Two order | 8.470 | 0.058 | 0.124 | 0.774 | 0.245 |
| FL | Third order | 9.390 | 0.079 | 0.262 | 0.094 | 0.724 |
| L X | Third order | 6.438 | 0.056 | 0.098 | 0.256 | 0.064 |
| R X | Third order | 9.846 | 0.083 | 0.741 | 0.061 | 0.561 |
| Vermis VI-IX | linear | 0.456 | 0.02 | 0.297 | 0.770 | 0.770 |
| Vermis VI | linear | 13.594 | 0.069 | 0.015* | 0.645 | 0.155 |
| Vermis VII | Two order | 3.766 | 0.027 | 0.838 | 0.839 | .0330 |
| Vermis VIII | linear | 0.501 | 0.005 | 0.750 | 0.531 | 0.636 |
| Vermis IX | Two order | 1.042 | 0.008 | 0.564 | 0.142 | 0.249 |
| Vermis X | Two order | 16.779 | 0.109 | 0.367 | 0.117 | 0.378 |

Total：Cerebellar volume, CM: Corpus Medullare, AL: Anterior Lobe, PL: Posterior Lobe, L: Left, R: Right. Age×Sex represents the interaction between age and sex. ** *p*<0.001, **p*<0.05.
