# Supplementary figures and images for: Morphological changes in the cerebellum during aging: evidence from convolutional neural networks and shape analysis
Source: Front Aging Neurosci. 2024 Apr 17;16:1359320. doi: 10.3389/fnagi.2024.1359320 (PMC11061448; doi:10.3389/fnagi.2024.1359320)

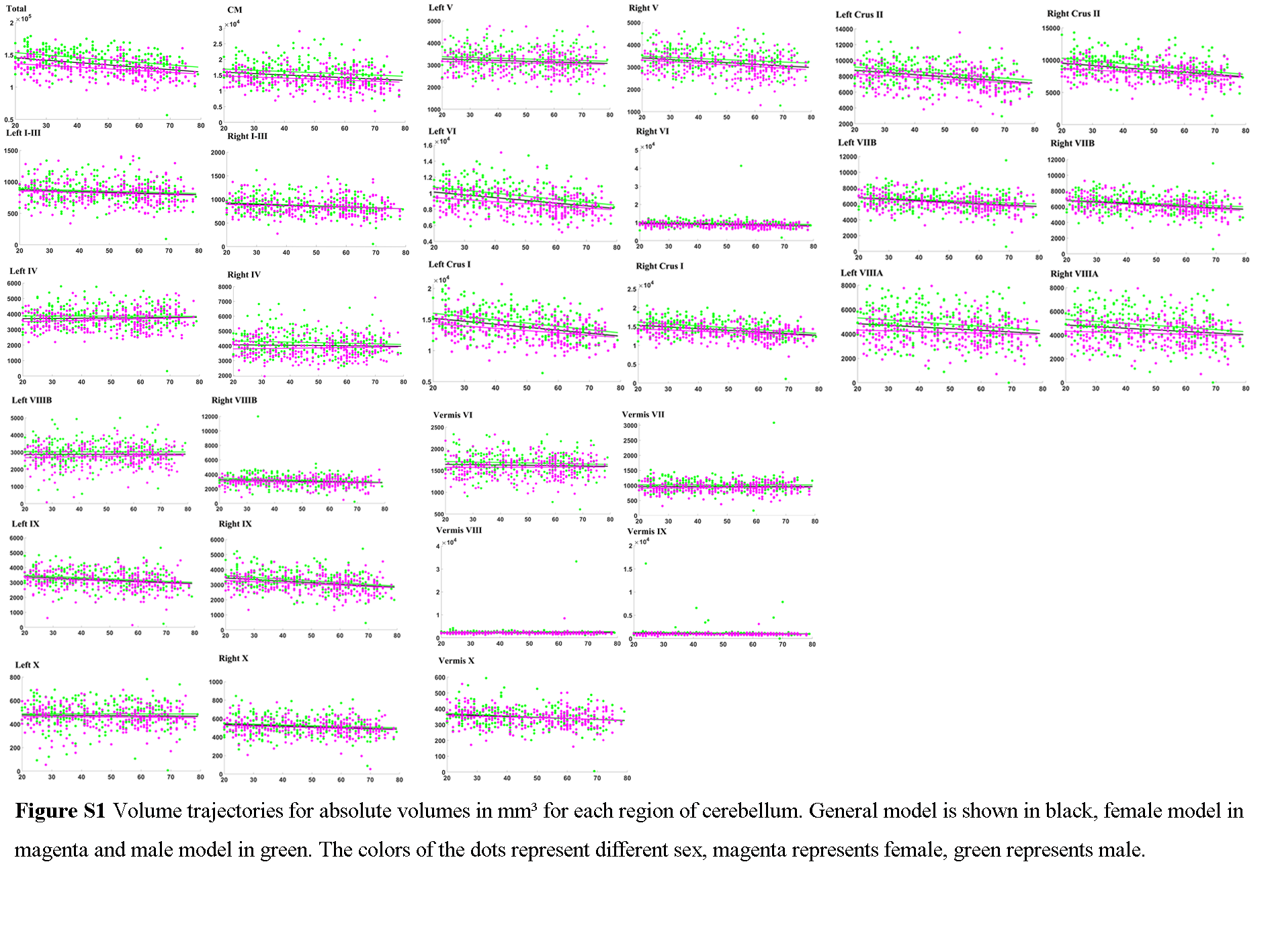

Supplement: Supplementary file 2 [file Image_1.TIF]
